# 1275. Dynamics of *Enterococcus faecalis* Cardiolipin Synthase Gene Expression Reveal Compensatory Roles in Daptomycin Resistance

**DOI:** 10.1093/ofid/ofab466.1467

**Published:** 2021-12-04

**Authors:** April Nguyen, Vinathi Polamraju, Rutan Zhang, Truc T Tran, Diana Panesso, Ayesha Khan, Eugenia Mileykovskaya, Yousif Shamoo, Libin Xu, Heidi Vitrac, Cesar A Arias

**Affiliations:** 1 University of Texas Health Science Center at Houston, Houston, Texas; 2 Univ of Texas Health Science Center at Houston, Houston, Texas; 3 University of Washington, Seattle, Washington; 4 Center for Antimicrobial Resistance and Microbial Genomics, UTHealth, Houston, TX, Houston, TX; 5 McGovern Medical School, Houston, TX; 6 UTHealth, Houston, TX; 7 McGovern Medical School, UTHealth, Houston, TX; 8 Rice University, Houston, TX; 9 Tosoh Bioscience, Philadelphia, Pennsylvania; 10 CARMiG, UTHealth and Center for Infectious Diseases, UTHealth School of Public Health, HOU, TX ; Molecular Genetics and Antimicrobial Resistance Unit and International Center for Microbial Genomics, Universidad El Bosque, BOG, COL, Houston, Texas

## Abstract

**Background:**

Daptomycin (DAP) is a lipopeptide antibiotic targeting membrane anionic phospholipids (APLs) at the division septum, and resistance (DAP-R) has been associated with activation of the *E. faecalis (Efs*) LiaFSR response and redistribution of APL microdomains (predicted to contain cardiolipin) away from the septum. *Efs* encodes two putative cardiolipin synthase genes, *cls1* and *cls2*. While changes in Cls1 are associated with DAP-R, the exact roles of each enzyme in resistance are unknown. This work aims to establish the contributions for both enzymes in the development of DAP-R.

**Methods:**

*cls1* and *cls2* were deleted individually and in tandem from *Efs* OG117∆*liaX* (a DAP-R strain with an activated LiaFSR response). Mutants were characterized by DAP minimum inhibitory concentration (MIC) using E-test and localization of APL microdomains with 10-N-nonyl-acridine orange staining. Quantitative PCR (qRT-PCR) was used to study gene expression profiles of *cls1* and *cls2* in *Efs* OG117∆*liaX* relative to *Efs* OG117. Membrane lipid content was analyzed using hydrophilic interaction chromatography-mass spectrometry (HILIC-MS).

**Results:**

*cls1* was highly upregulated in stationary phase concurrent with a decrease in *cls2* expression. However, independent deletion of *cls1* or *cls2* in the DAP-R background resulted in no significant phenotypic changes from the parent strain. Interestingly, qRT-PCR showed that *cls2* expression was upregulated upon deletion of *cls1* (and vice-versa), suggesting a compensatory role for one enzyme upon deletion of the other (Fig 1). When comparing membrane lipid content between *Efs* OG117∆*liaX*∆*cls1* and *Efs* OG117∆*liaX*∆*cls2,* there were no significant differences in both the overall amount or species of cardiolipin generated, further supporting a potential redundancy between the cardiolipin synthases (Fig 2). Ultimately, double deletion of both *cls* genes lowered the DAP MIC relative to the parent strain and restored septal localization of APL microdomains.

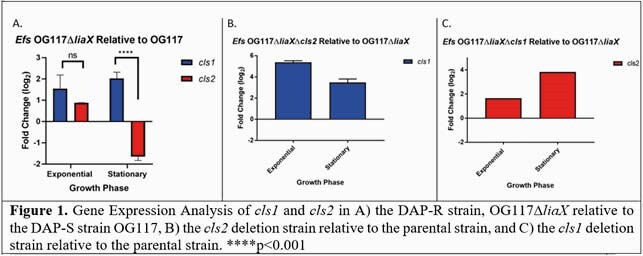

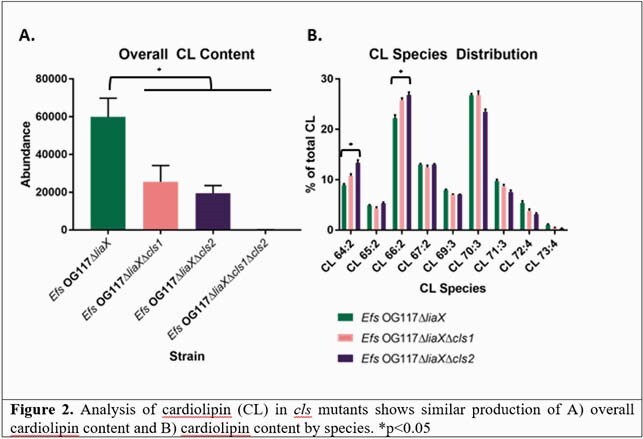

**Conclusion:**

Overall, Cls1 has a predominant role in the development of DAP-R in *E. faecalis*. However, here, we describe a novel compensatory role for Cls2 under conditions in which there is no functional Cls1 to maintain the DAP-R phenotype.

**Disclosures:**

**Truc T. Tran, PharmD**, **Merck** (Grant/Research Support) **Cesar A. Arias, M.D., MSc, Ph.D., FIDSA**, **Entasis Therapeutics** (Grant/Research Support)**MeMed Diagnostics** (Grant/Research Support)**Merk** (Grant/Research Support)

